# [Corrigendum] Stormorken syndrome caused by *STIM1* mutation: A case report and literature review

**DOI:** 10.3892/mi.2023.122

**Published:** 2023-11-20

**Authors:** Wenqiang Sun, Jinhui Hu, Mengzhao Li, Jie Huo, Xueping Zhu

Med Int (Lond) 2:29, 2022; DOI: 10.3892/mi.2022.54

Following the publication of this article, an interested reader drew to the authors’ attention that, in Table I on p. 4, the entry for ‘c.1095G>C(p.K365N) (1)’ should not have been featured under the ‘STIM1 EF-hand (N=46)’ column (i.e., the first column in the Table). The authors regret the statistical error that was made here, and were able to verify from the original reference (Jiang LJ, Zhao X, Dou ZY, Su QX and Rong ZH. Stormorken syndrome caused by a novel STIM1 mutation: A case report. Front Neurol. 12: 522513, 2021) that the entry for ‘c.1095G>C(p.K365N) (1)’ should have been included under a separate column for ‘STIM1 ORAI1’.

The authors are grateful to the Editor of *Medicine International* for allowing them the opportunity to publish this corrigendum, and thank the reader for drawing this matter to their attention. All the authors agree with its publication, and also regret any inconvenience that this mistake has caused.

